# Clinical Lectures Delivered by Professor Chomel at the Hôtel Dieu in Paris, (Typhoid Fever)

**Published:** 1836-07

**Authors:** 


### Art. II.

*Leçons de Clinique Médicale, faites à l'Hôtel-Dieu de Paris par le Professeur* A. F. Chomel, *recueillies et publiées sous ses yeux par* J. L. Genest, d.m.p. (*Fièvre Typhoide*). Paris: 1834. 8vo. pp. 548.

*Clinical Lectures delivered by* Professor Chomel *at the Hôtel Dieu in Paris,* (*Typhoid Fever.*) Collected and published by J. L. Genest.

M. Chomel is one of the clinical professors appointed by the School of Medicine of Paris, and is better known as a teacher than an author; for, although he has published two works, one of which went into a second edition, yet, for the last ten years, he has apparently devoted his time almost exclusively to the oral instruction of his pupils. His reputation among foreigners is consequently less than that of inferior men, who have taken the more effectual way of extending their names beyond the confines of their own country. Those who have been his pupils, however, hold him in high estimation. His unwearying attention to his hospital patients, his careful examination of every case, although his ample experience might justify more haste, the untiring assiduity with which he displays to his class the recent specimens of diseased structure which examinations after death afford, mark a devotion to his profession, and a regard for the interests of his pupils, which cannot fail to command their respect; whilst his powers of eloquence and clear arrangement as a lecturer call forth their admiration of his abilities.

This volume contains the substance of M. Chomel's clinical lectures on Fever, arranged under his own inspection by M. Genest, a former pupil. M. Chomel regards all fevers as essentially the same; constituting one genus, but capable of division into several varieties. He adopts the name Typhoid Fever, which is given to the continued fever prevalent in Paris, and of which this volume may be considered as the history. After describing its general symptoms, he enters minutely into its complications. This simplification in the nosology of fevers is based on the discoveries of pathological anatomy, and is well calculated to clear away many difficulties with which the French nosologists had encumbered a subject in itself sufficiently intricate. The inflammatory, bilious, ataxic, and adynamic fevers of Pinel,—the entero-mesenteric fever of Petit and Serres,—the "exanthème intestinal" of Andral,—the "dothinentérite" of Bretonneau,—the "ileo-ylidite" of Bally,—and the "entérite folliculeuse" of others, are thus considered as typhoid fever, modified by certain circumstances. In this country, the general custom is to apply the name typhus to fever attended with great prostration of strength: when the symptoms are milder, it is called simply continued fever, or subdivided into synochus and synocha, if the practitioner adheres to the nosological system of

Cullen. It is to be understood, that the term typhoid fever here used would be applied by M. C. as including all these cases. This classification, which is becoming the favorite one among the French pathologists, is, we trust, finding favour also in this country. The division so long adopted by Cullen cannot be applied at the bedside; for the same case may at different periods put on the three different forms, and oblige the systematic adherent to this classification to change repeatedly the name of one disease. The great object formerly was to investigate the causes rather than the phenomena of fever, and the treatment which was recommended depended on the theory of the disease; varying, of course, according to the views of different schools. In the investigation of fever in this and in the more recent works of pathologists, the attention is particularly directed to the lesions discovered after death, and an endeavour is made to connect these, as far as possible, with the signs during life. The great practical advantage of this latter plan is to destroy devotion to any one system, and to enforce the necessity of minutely attending to the symptoms of every case, and acting in accordance with the information so obtained. There is some danger of this leading to the opposite extreme, of regarding the general symptoms of fever as the expression of some local disease. It has not, however, had this effect on M. Chomel, who regards fever as a general disease of the whole system, whilst he fully recognizes its local complications.

The plan which will be pursued in this article will be to give a condensed analysis of M. Chomel's volume, in order to furnish a complete history of the typhoid fever of Paris. The mere translation of well-selected extracts from a work on Fever would be of little use to any reader.

*Symptoms and Progress of Typhoid Fever.* Although the forms of fever differ greatly, yet there are a series of symptoms common to all, which will be first stated.

1. Precursory stage, and invasion.

In the great majority of cases the attack is sudden. Thus, in 73 out of 112 cases, there were no premonitory symptoms observed. We would here remark, that this statement should be taken with some little reservation; for these were all hospital patients, whose accounts cannot in such matters be implicitly relied on, from their want of accuracy in relating the history of their diseases, and a deficiency in the power of observing even their own feelings. Medical men, who are aware of the importance of close observation, and who have suffered from fever, have detected, in their own cases, some deficiency of mental vigour preceding the rapid development of the fever itself; and such cases among the poor would be at once set down as instances of sudden invasion without prelude.

The premonitory symptoms are—depressed expression of countenance; diminished power of application to intellectual employments; debility; anxiety; a feeling of uneasiness or discomfort; pain in the limbs; loss of appetite, foul tongue, nausea, &c.

The first symptom of invasion is generally intense headach on waking in the morning; sometimes, though rarely, preceded by diarrhœa. The expression of countenance becomes rapidly altered; a marked appearance of stupor is not unfrequently observed from the first. Rigors followed by heat; great muscular debility, so that the patient takes immediately to bed, or, if he attempts to walk, staggers as if drunk. On asking such patients how they came to the hospital, they almost constantly reply that they were carried in. Diarrhœa is one of the most constant early symptoms, together with abdominal pains. M. Chomel divides the course of the disease into three periods, of a week each, as, in the most simple cases, there are a peculiar set of symptoms proper to each of these periods.

This division must not be regarded as any thing more than an approach to a natural one, as it will be seen hereafter that the period of termination of the affection follows no fixed times. It is, however, a convenient division, and, if looked upon merely in this light, it cannot be objected to.

*First Period.* The change in the physiognomy is striking: it is expressive of apathy, from which the patient cannot be roused except by questioning him in a manner to excite strongly his attention. His answers show that his intelligence is diminished. Muscular debility causes him to lie in one position on his back. Constant wakefulness, or dreams so vivid that he confounds them with sensations, and believes that he has not slept. Headach, generally confined to the forehead, continues with more or less intensity during the whole period. The secretions of the mouth become thick and glutinous, so that the finger, if applied to the tongue, is detached with some little difficulty. The tip and edges of the tongue are red, with a small white border on each side; sometimes preceded by a whitish or yellow coating, with a foul taste. As the mouth dries, the whole mucous membrane becomes of a uniform red colour, the lips crack, and the teeth look brilliant from the dried layer of mucus which covers them. Anorexia, sometimes with nausea and vomiting; dysphagia; great thirst; diarrhœa almost universally; from four to eight daily evacuations. In many cases some gaseous distention of the intestines may be detected by percussion, even before there is any enlargement sensible to the eye. A gurgling sound is heard when the lower part of the belly, and particularly the right iliac region, is pressed with the hand. This, which is owing to the presence of air and liquids in the intestines, and may be also connected with a pathological condition of the ileo-cæcal valve, is as rare in other diseases as it is common in this: it is more frequently present in the second and third periods. In the majority of cases there is increased sensibility over the bowels on pressure, rarely acute, and often not complained of, unless strong pressure is employed: it may be confined to the right iliac region, to the whole hypogastric or epigastric regions, or it may extend over the whole abdomen; in which case, it is often connected with a painful state of the neighbouring parts, as the chest, or even the whole surface. During the first days there is generally strong reaction of the circulation, with marked inflammatory symptoms: pulse large, and sometimes resisting and frequent; skin red. Towards the end of this period these symptoms diminish; the pulse may be more rapid, but it is soft; the skin, which was covered with abundant acid perspiration, becomes dry and hot. Urine scanty, high coloured, and fœtid; early epistaxis is common, and a valuable diagnostic symptom. The bleeding is rarely copious, but often occurs several times. The state of the lungs is important in diagnosis: often from the first there is a general sibilant râle over both lungs, more marked inferiorly and posteriorly. The cough is rarely in proportion to the râle; the expectoration is scanty, viscid, and transparent. Obstruction of the nostrils by dried mucus or blood, and extreme meteorism, often produce dyspnœa. In some few cases none of these symptoms are present during the first week, except a febrile condition. Death rarely occurs during this period. Of forty-two fatal cases, only one death took place in the first week.

*Second Period.* The eruption which is peculiar to typhoid fever usually appears between the seventh and ninth days. It consists of small rose-coloured spots, disappearing on pressure, from half a line to two lines in diameter, round, not or hardly elevated; scattered over the abdomen, sometimes on the breast, more rarely on the thighs, arms, and forearms: their number varies; in order to be characteristic, there should be at least fifteen or twenty; no value can be attached to two or three. They do not all appear at the same time; their duration is uncertain; they ordinarily disappear in two or three days, in other cases they remain twelve or fifteen days, but then it is probable there are successive eruptions. Out of seventy cases occurring in 1830-1-2, where attention was paid to this point, there were only sixteen in which the eruption did not appear. Of these fifty-four cases presenting the eruption, there were none in which it appeared before the sixth day, and in two cases it appeared as late as the thirty-sixth day of the disease. This agrees with the results of a larger number of cases examined by Louis. The value of this sign in the diagnosis will be evident, when its frequency in typhoid fever is contrasted with its infrequency in other acute diseases, and that, when it does appear in the latter cases, it is never abundant. This eruption is distinguished from petechiæ and fleabites by its colour disappearing entirely under pressure, and returning as soon as it is removed. Hildenbrand observed a similar eruption in the typhus of camps; and, in 1814, M. Chomel had an opportunity of verifying his observation in Paris. The extent of this eruption in some epidemics gave rise to the term petechial fever. Sudamina are sometimes observed at a later period, but they are not so intimately connected with this disease as the eruption just described: they are small, demi-hemispherical, transparent vesicles; when viewed obliquely they have a brilliant appearance, but, when looked at perpendicularly to their axis, they escape observation. This explains why they have been so rarely mentioned by authors. They are readily distinguished by the touch: they appear at first on the sides of the neck, and in the folds of the armpit and groin, from thence in some cases extending to the trunk and limbs. It is a symptom of some importance, as it is much more frequent in this disease than in any others with which it may be confounded. Sloughing may be produced by pressure, as on the sacrum and heel, and also on the back of the hairy scalp, where it is often overlooked; it sometimes occurs spontaneously and suddenly, as on the inner surface of the thighs or upper part of the foot; it may follow the application of sinapisms or the irritation of urine and fæces. In the least serious cases, which are also the fewest, the debility and stupor (if present) remain in the same degree as in the first period; but, in the most serious forms, the prostration of strength is complete, and the patient lies on his back, an inert mass. The muscles of the throat sometimes partake of the debility, and deglutition becomes impossible; the liquids being rejected through the mouth or nose. Dysphagia may depend on inflammation about the epiglottis, or ulceration of the mucous lining of the fauces and œsophagus. Involuntary discharge of stools is another symptom of muscular debility. There may be retention of urine, producing, if overlooked, paralysis of the bladder. If the respiratory muscles share in the deficiency of muscular power, the patient is in danger of suffocation. Together with this extreme prostration of muscular force, it is not uncommon to find subsultus of the tendons of the arms and hands, or convulsive twitches of the nose and upper lip, and carpology. General and permanent rigidity of the limbs is almost always a fatal symptom: a boy of fifteen, however, recovered, who had this symptom for two days together, with aphonia and remarkable smallness of the pulse. In mild cases the headach ceases, and, instead of constant wakefulness, the patient is in a drowsy state, from which it is impossible to rouse him, except for a few moments. This is the Coma somolentum of authors, and often lasts many days. When this stupor is so great that the patient cannot be roused by any excitement, he generally dies in a few days in the same state. Instead of stupor, some have delirium, coming on in the evening or at night, or constant; either violent or tranquil. Deafness, in most cases independent of stupor, is very frequent. Occasionally the senses of sight and taste are weakened. The force of the general febrile symptoms is much diminished; the pulse is small, weak, trembling, jerking, or intermittent; generally from 100 to 120; in others, from eighty to ninety; in a few, it falls as low as forty or fifty at the termination of this period. There is an increase of fever in the evening, sometimes hardly perceptible, at others violent, and more rarely preceded by rigors, and terminating by sweating, than in the first period. Skin of a more acrid heat, drier and rougher; thirst less urgent; the nostrils having become impervious to air, the patient breathes wholly through his mouth, and the mucus covering it becomes dry, and changes from a brown colour to a brilliant black: this has been mistaken for effusion of blood. Diarrhœa continues; somtimes less frequent. Hemorrhage from the bowels, which, if abundant, speedily destroys the patient: this symptom is important in the diagnosis, as it is much more common than in other diseases. Meteorism continues or increases; abdominal pains not complained of, except in the mildest cases; respiration often more difficult, although the local symptoms do not increase; the breath and perspiration have an offensive smell, peculiar to fever. In mild cases the progress is much more simple, so that the febrile state, with some few only of the characteristic symptoms, mark the nature of the disease. Of forty-two fatal cases, nine deaths took place during this stage.

*Third Period.* The symptoms either improve and lead to convalescence or become aggravated, terminating in death. Thus, thirty-two patients, out of forty-two who died of fever, died in this stage; and, of ninety cases of recovery, convalescence commenced in one only during the first period; and, when the symptoms were severe, there were no instances of improvement before the end of the second. Improvement of expression, and attention to what is passing, are often the first stages of amendment; or the comatose state is exchanged for peaceful sleep, on waking from which the patient partly recovers his intelligence: he is able to move himself a little; the tongue and mouth become moist; meteorism diminishes; the evacuations are of a more yellow colour, less fluid and fœtid. Sometimes, at the moment when the first amendment of the symptoms commences, solid and formed stools are passed; sometimes black, dry, and in prodigious quantities: they had probably lain hidden in the cells of the colon. The patient becomes aware of the passage of his evacuations; respiration more free; expectoration less viscid; pulse slower, less soft, and firmer; skin more supple, or moistened. At this period it is not unusual for abscesses to form in parts of the body which do not appear to have been irritated. The face becomes thinner, and the features and expression more marked. Of sixty-eight favorable cases, the convalescence commenced by one or more of the previous symptoms on the following days:

In 1 patient, the 8th day after the attack.
1 — 9th.
4 patients, the 12th.
3 — from the 12th to the 14th days.

| | | |
|---|---|---|
| In 10 | patients, | from the 15th to the 16th. |
| 15 | — | from the 17th to the 20th. |
| 14 | — | from the 21st to the 25th. |
| 11 | — | from the 26th to the 30th. |
| 8 | — | from the 31st to the 40th. |

It will be seen by this table, although the days on which the improvement commences are very variable, yet that, in fifty cases out of sixty-eight,—that is, nearly three out of four, the improvement commenced from the fifteenth to the thirtieth day.

In fatal cases, the stupor augments, expression more changed; the mouth is drier, or, if moistened, it is only by the secretion of grey, viscid mucus, mixed with blood, and fœtid. Respiration more difficult, stertorous; sometimes, towards the last days, crepitation is heard posteriorly and inferiorly, which is replaced by complete absence of respiration. Pulse more feeble; heat diminishes; skin dry, covered with cold, glutinous sweat; emaciation general and rapid; eyes hollow; features drawn down with a fixed expression, (facies Hippocratica.) If the patient can speak, it is with difficulty and with a trembling voice; the answers are unintelligible, even if the words are understood. The exhaustion of strength is complete, and the comatose debility is speedily followed by death. In some few cases, either at this period or during convalescence, the patient is suddenly seized, if his sensibility is sufficiently excitable, with extremely acute pains in the abdomen, sensation of sinking, alteration of expression, nausea and vomiting, and the symptoms of typhoid fever give way to those of partial or general peritonitis. The pulse is small and thread-like, the abdominal pains are excessive. This sudden attack of peritonitis, depending on no apparent external cause, is owing to perforation of the coats of the intestines and effusion of fæces into the peritoneal cavity. It is almost inevitably fatal. Two out of forty-two fatal cases died from this cause. Erysipelas of the face is a very fatal complication: it was observed in four cases out of 130, and all four died. The convalescence from fever is not rapid; in some cases it is extremely prolonged. Satisfying the appetite, which is often voracious, frequently leads to very serious consequences. Œdema of the lower extremities sometimes follows fever; so does mental derangement in some few cases, but it generally disappears when the patient resumes his previous habits of life.

Such is a brief sketch of a very full history of the common symptoms of typhoid fever, as seen in the Paris hospitals. The method and arrangement of the whole is highly judicious, whilst the description is clear and comprehensive. The minute attention to symptoms is seen in the description of the peculiar eruption of rose-coloured spots, the gurgling sound on pressure of the abdomen, the discovery of gangrene on the back of the head, &c. To such points we would beg the attention of practitioners in this country, who have, from situation and circumstances, constant opportunities of observing fever in all its forms. The eruption of red spots is almost universally present in the typhoid fevers of Paris, and is one of the points to which the physicians of the hospitals particularly direct their attention as a diagnostic mark.

In this country, these "taches rosées" are, if observed, frequently confounded with others under the term Petechiæ; but there is an essential distinction between the typhoid eruption, which disappears on pressure, and consequently has the character of an erythematous eruption, and spots which depend on an effusion of blood beneath the skin, which are not influenced by pressure, and which Willan called Purpura. The one is an eruption, the other a hemorrhage; and, as they probably depend on two very different states of the system, and are of very different value as regards the prognosis, it is in every respect important to distinguish them. We suspect that the little notice which has been taken of this eruption in this country is owing rather to the want of observation than to its non-existence; for the appearance is so inconspicuous, that, unless the attention of the examiner had been directed to it by another, or had had the opportunities of comparing together numbers of cases at the same time, and thus of deciding on the essential and non-essential symptoms for himself, he might very probably give it no attention, although he had observed it, from considering it to be a trifling and accidental complication. M. Chomel, in stating the proportion of cases in which he observed it, does not go back more than three years; as it was only since that time that his attention was especially called to it, although he had attended to the petechiæ of camp-typhus twenty years ago.

The description of the appearance of scybalæ in the stools at the commencement of the recovery, is an example of the peculiar view which our neighbours take of the causes of symptoms. In this country we should most probably attribute the amelioration of the symptoms to the discharge of the accumulated fæcal matter: M. Chomel regards it as a symptom of commencing convalescence, that is coincident with improvement in other respects, and in no wise as producing it. This difference is owing to the different points of view from which the same subject is looked at: we are engrossed in treatment, and see in this symptom only an effect which we could and should have produced much earlier, and believe that its production even at that time would have been followed with good effects: our neighbours, who are devoted to diagnosis, and look at the powers of nature with more respect and confidence, regard it merely as one of the changes which mark the power of nature in relieving herself from the disease. In a practical point of view, there can be little doubt that the presence of these scybalæ must have been a source of irritation to the patient, and that, if medicine had produced the same effect earlier which nature did eventually, the symptoms would have been milder, although the course of the disease would not have been cut short.

The negative evidence of such writers as M. Chomel on crises and critical days is more valuable, from their being great believers in the efforts of nature in the cure of fever, although not professedly humoral pathologists. Of ninety-four cases, there were two in which copious perspiration was followed by benefit; and two others where an abundant discharge of fæcal matter coincided with an amelioration of the symptoms; in the remaining ninety, nothing similar was observed, so that these four cases can only be regarded as rare exceptions. In some few instances improvement preceded evacuations, which have been called critical: they were proofs rather than causes of amendment. The only phænomena which really appeared to precede improvement were abscesses, in six cases out of eighty. The list given of the days in which improvement took place shows that it happened many times in each day between the fifteenth and thirtieth. These observations do not tend to support either the doctrine of critical days or of critical evacuations; and, as the cases have been collected and examined in the most careful way, the results must be admitted as correct as far as relates to the fever which is described. Cullen was disposed to think that the fault lay in the physician who denied the doctrine of crises in fevers; but this objection cannot apply to such observers as M. Chomel. The accurate way in which the examination of the question is conducted justifies the conclusion that the phænomena did not occur in a large number of cases, and therefore the evidence must not be regarded as a mere negative assertion that certain circumstances were not observed.

The apparent discrepancy between the assertions on these questions of former writers of credit, and the results of facts which come more immediately beneath our notice in our own times, admit probably of explanation, if it is allowed that fevers are so modified by circumstances, that hardly two epidemics are precisely similar. That the notions of crises and critical days were sanctioned by many humoral pathologists without enquiry, from its agreeing with their favorite theory, is very probable; but we cannot doubt that the opinions of Hippocrates and men of like stamp were founded on accurate observation. The diseases of the Greeks, however, must have differed materially in character, progress, and termination, from those which affect the nations of modern Europe, inasmuch as external circumstances influence the pathological as well as the physiological actions of the body. "There is," says Dr. Latham, "in healthy and vigorous bodies, a certain regularity and balance of function, which, even when disease befals them, is seldom lost, but their morbid action is still harmonious and proportional. In them diseases are often severe, but they are generally simple. On the other hand, the weak and valetudinary, who at the best are full of jars and incongruities, are obnoxious to the strangest forms of disease, hard to understand, and hard to treat." As physical perfection was most highly prized by the Greeks, the preservation of vigorous health was the object of their most assiduous care. Their civil and military institutions required and favoured exercises and manners best adapted to develop their bodily powers, whilst the climate allowed their form to grow unfettered. The weakly constituted probably perished in their infancy, by that barbarous custom which gave the parent the power of putting to death his infant children. That this practice, which was so universal that their philosophers did not venture to disapprove of it, would be put into execution on sickly children, cannot be doubted in a nation where delicacy of organization was considered a reproach. Among such a people the progress of disease must have been but little complicated, and the curative efforts of nature could have met with few obstacles to their free exercise. If fevers were ever relieved by critical evacuations, and followed a course which would justify the fixing of critical days, it must have been among such a people.

The enumeration of the symptoms is followed by a detail of the morbid changes which are discovered after death. This occupies nearly half of the volume, the changes being described with the utmost minuteness, and an attempt made to elucidate the order in which they happen. Cases illustrate the whole.

The researches of recent pathologists have shown that there are some lesions constantly, or almost constantly, found; whilst others are less frequently met with: hence they may be divided into the constant and the accidental.

1. *Constant lesions.*

These include diseases of the follicles of the intestines and of the mesenteric glands, which are almost always discovered. The follicles of the intestines are either isolated or in groups: the first are called the glands of Brunner, the second of Peyer. The earliest period from the commencement of fever at which M. Chomel has had an opportunity of examining their morbid condition, was in a case in which death took place on the seventh day. Out of fifty-five cases examined by Louis, the most recent was on the eighth day. At this period the intestines are distended with gas, which increases their transparency, and shows externally a number of opaque spots, of various sizes, along the curvature of the small intestines. On examining these internally, they are found to be produced by the deposition beneath the mucous membrane of a yellowish white matter, rather friable, in the situation of the follicles, giving the aggregated glands of Peyer the form of a patch, generally elliptical, varying from three inches to one inch in the longest diameter; and the isolated follicles of Brunner that of a large pimple, which some pathologists have mistaken for pustules. Their colour varies according to that of the neighbouring parts: if the mucous membrane is pale, the patches are of a dead white; and if red, they are of a much deeper red. The larger patches are found in the ileum and termination of the jejunum; most numerously towards the end of the small intestines; and, when but few exist, they are almost immediately above the ileo-cæcal valve, the ileal surface of which is sometimes covered. They are almost always opposite to the attachment of the mesentery. The enlarged isolated follicles are in some subjects much more numerous, and are scattered indiscriminately; they rarely are found above the last third of the ileum. In one-third of the cases, the isolated follicles of the large intestines are diseased; those in the colon are much larger than in the ileum, and they again diminish in size as they approach the rectum. The mesenteric glands which are nearest to these enlarged follicles become enlarged, and of a deeper colour. Sometimes their consistency is firm, at others soft. The disease of the follicles appears to commence in those nearest the ileo-cæcal valve; and, as the fever proceeds, those higher up in the small intestines are implicated: the affection of the mesenteric glands follows the same course. The number of diseased patches varies from one to twenty or thirty. The isolated glands are often free from disease, but in these cases death has generally taken place from the fifteenth to the twentieth day. When death had occurred earlier, they were found very numerously in the lower part of the ileum. If the patient dies during the second period of fever, other changes in these enlarged follicles are observed. Ulceration takes place, commencing either in the mucous membrane covering the patches, which it gradually destroys, or the layer of yellowish matter is softened primarily, and the mucous membrane is consecutively removed. In both cases the ulceration of the mucous membrane is the result of the diseased state of the follicles, as it only commences over these patches, and is confined during this period to these parts. This was the case in forty-two instances of death during this stage, without any exception. In ninety-two cases closely observed by MM. Chomel and Louis, ulceration commenced from the eighth to the twelfth or fifteenth days from the first attack. The ulceration proceeds from the ileum upwards. The isolated follicles more rarely ulcerate: in one case only out of forty-two were they alone ulcerated. In some cases during this second period, the mucous membrane covering these patches becomes of a dark colour, separates from the subjacent tissues, and is observed to be perforated with a large number of holes, giving it a reticulated appearance: these holes are the orifices of the enlarged follicles. Beneath this the submucous tissue is found, or a thin layer of the white deposit. The separation of this reticulated mucous membrane exposes an ulcerated surface.

If the subject dies at a later period, new changes are observed. Sometimes there is no trace either of the ulcerated or reticulated patches, but merely ulcers, whose edges have no trace of the debris of the whitish deposit, and whose form and situation may not indicate their origin in the glands of Peyer. The ulcers may be not elliptical, but round, or very minute, or an ulcer of two inches in breadth may occupy the internal circumference of a portion of intestine, the edges not being irregular, but as if cut with a stamp. The question is important whether or not these ulcers have their origin in the diseased follicles? When there are traces of the debris of the whitish matter on the edges of any of them, there can be no doubt as to their nature. If, after a typhoid affection, ulcers are found in the situation and of the form of the follicles of Peyer, their origin is equally clear; if the situation and figure differ, it must be remembered that there are patches of shapes and sizes intermediate between the more common oval ones, and that these alone may have ulcerated. The symptoms will throw light on their nature. Those cases alone can be doubtful where the symptoms of typhoid fever were obscure: here the variable forms of fever should be borne in mind, and, if the length of the disease agrees with that of typhous fever, the ulcers must be attributed to disease of these follicles. An acute disease arising in a person in perfect health, and terminating fatally in a few days, in which ulcers were found in the intestines, would be an exception; but M. Chomel has never seen such a case. In all those cases where ulcers have been found, the disease has lasted at least eighteen days. M. Chomel thinks himself justified in concluding, until new facts are brought forward which may modify his inferences, that ulcers which are formed in the intestines after an acute disease are the result of lesions of the follicles, and not a primary affection of the mucous membrane.

It seems probable, from the form of the ulcers, that in some cases they extend beyond the original situation of the follicles.

There are two principal varieties in the ulcers; some being simple, the others connected with hypertrophy of the cellular and muscular coats. In the first, the mucous membrane forming the edges is white and thin; neither red, softened, nor thickened: almost on a level with the ulcerated surface; with no appearances of inflammation; the shape is regularly rounded. In the second variety, the edges are prominent, deeply cut, more or less of a slate colour; whilst the cellular and muscular coats are much thickened; the bottom of the ulcer is sometimes formed by the thickened muscular coat, or, if that is destroyed, by the peritoneal coat only; whilst the edges are three or four lines in thickness, from the hypertrophied state of the mucous, cellular, and muscular coats. When perforation of the intestine takes place, it is generally by a small hole, always corresponding to an ulcer, and generally in the last foot of the small intestines. Around the perforation are found adhesions or purulent matter; and in this effort of nature to produce adhesion rests the only hope of cure. Perforation occurs from ulceration or mortification of the peritoneal coat; it may also be favored by the intestines being distended with air, and perhaps to this cause may be attributed the fact that perforation occurs much more frequently in fever than in phthisis, where ulceration is very frequent, but meteorism rare.

*Cicatrization of Ulcers in the Intestines.* If death takes place at a later period than those previously considered,—that is, six weeks, two months, or even later, after the attack,—the edges of the simple ulcers are still more flattened; so that often, in several points, it is difficult to distinguish the sound membrane from the ulcerated surface: in the centre are small inequalities, which seem to answer to the granulations of common ulcers, but are distinguished with difficulty. If death ensues at a later period, no traces of ulceration sometimes can be found, or the spots where ulcers did exist may appear more smooth, or darker, or slightly depressed. Facts are wanting to enable us to specify the time when all traces vanish. For many years M. Chomel has examined the intestines of those who have died of other diseases, and yet he has never found traces of cicatrices, although many of these, from their own accounts, had suffered from typhoid fever previously. This is to be explained by the colour and appearance of an internal cicatrix approaching much nearer to those of the surrounding parts than an external one.

When no ulceration or destruction of the mucous membrane has taken place, it appears that resolution of the diseased follicles may happen. Thus, in patients who have died when some patches have ulcerated, other patches which have not ulcerated are less elevated than in those cases where death has taken place earlier; and, again, there is a still further diminution in the elevation of these patches in still more protracted cases. The absorption, as might be expected, seems to follow the same course as the deposition, being earlier in the patches near the ileo-cæcal valve. In a few cases, either the whole or parts of the patches, during probable absorption, have been found of a deep blue or slate colour, and even black. The isolated glands are more rarely coloured. This should not be confounded with a more frequent appearance of both kinds of follicles, in which their little orifices are surrounded by a minute black circle, giving the patches the appearance of a chin when its black beard is just shaven. M. Chomel considers this to be a normal condition in such individuals. The enlarged mesenteric glands in those cases, where cicatrization goes on, diminish in size almost to their natural standard; but they become firm, red, violet, or even black, both externally and internally. They never ulcerate.

There are three other diseases in which diseased follicles have been discovered.

1. Cholera. In the greater number of patients who died of cholera in 1832, the follicles of the intestines were enlarged. The elevation was about the fourth or fifth of a line, of a darker colour than the surrounding parts, sometimes of a dull white, or red, or dark brown. They differed from those in typhoid fever,—1, in being much less elevated; 2, in the uniformity of the lesion at all epochs of the disease: there was no difference, although some patients died twenty-four hours, and others thirty-six days, after the first seizure. Ulcerations were never found. Both kinds of follicles were enlarged.

2. Phthisis. The resemblance between the diseased follicles in phthisis and in typhoid fever is more exact. In most subjects who die from phthisis, the isolated follicles are increased in size, and filled with a firm whitish substance, whilst others are ulcerated; thus approaching very nearly the change which takes place in typhoid fever. The distinguishing difference, however, depends on the changes in the glands of Peyer. Instead of a uniform layer of whitish deposit, the patches present on their surface a small number of tumours, exactly like isolated tubercular follicles: in some cases a few only have ulcerated, in others there are extensive ulcerations, with a few of these tubercles on the edges. Where there are extensive ulcers only, the induration and thickening of all the surrounding tissues distinguish their nature. The mesenteric glands, too, are converted into tubercular matter.

3. Scarlatina. The follicles are enlarged occasionally, as in cholera.

We have thus given, at some length, M. Chomel's description of the changes which the follicles of the intestines undergo in fever, as the importance of examining the intestines carefully in this country has been very generally overlooked. Within the last few years, M. Chomel has examined forty-two fatal cases of fever at the Hôtel Dieu, and there was not one amongst them in which these follicles were not more or less diseased. These facts are not novelties, but are merely corroborative of the statements of preceding observers. For many years morbid anatomy has been ardently cultivated in France, but it is only since MM. Petit and Bretonneau directed particular attention to this lesion that the intestines have been examined with that constancy and minuteness which were necessary to confirm the connexion between typhoid fever and this change of structure: and, although we may hold in little estimation the theories built upon this basis as to the nature of fever, yet we cannot but look at the facts themselves as of great importance in elucidating the history of the disease, and thus as likely to furnish surer guides to its treatment. Such discoveries show the importance of the principle so laboriously practised by M. Louis, of minutely examining all the organs of the body, and not only those where disease was suspected.

The importance of establishing the follicular origin of the ulcerations of the mucous membrane in fever, is more felt in France than in this country, as Broussais had converted fever into mere gastro-enteritis, and had based his treatment on his theory. The constant presence of ulcers in these cases gave a plausibility to the idea that fever depended on a local inflammation; but, by tracing the origin of these ulcers to enlargement of the mucous glands by the deposit of a white cheesy matter in their substance, and a subsequent sloughing of the mucous membrane covering them, whilst that around was in a natural condition, the theory of the inflammatory school was overthrown by their own weapon, morbid anatomy. Rational views of treatment, deduced from this knowledge, would correspond more nearly with those of our older physicians than with those moderns who imagined that fever could be cured by means of a few leeches, iced enemata, and slops.

The next point which M. Chomel endeavours to solve is the *Connexion between the Symptoms and the diseased Changes in the Follicles.* Headach was absent in one case only out of forty-two fatal cases. As it is the first symptom, it cannot be supposed to be the consequence of lesions which it precedes; and, as it does not increase gradually, at least in the majority of cases, but after a few days diminishes, its course is contrary to that of the disease in the follicles. Stupor is an important symptom: it is essentially adynamic, as it is always connected with prostration of strength. Two opinions have been urged as to its origin: the one that it was a late symptom, coming on when the constitutional powers were becoming exhausted; and the other that it was the effect of ulceration of the mucous membrane, and the absorption of pus, &c. into the system. That neither of these opinions is correct will appear from the facts that, in four cases out of twenty-nine in which stupor was present, it commenced before the eighth day, that is, before ulceration; and in twenty-three of these it was well marked many days before the eighteenth day, on which, as a rule, ulceration begins. Acute delirium does not appear to be connected with this change. Its connexion with diarrhœa is a more important enquiry. In forty cases out of forty-two there was diarrhœa; thirty-four out of forty had it on their entrance, and six after their admission into the hospital; and in these six it commenced on the fifth, sixth, seventh, thirteenth, sixteenth, and twenty-first days. M. Chomel infers from these data, "that diarrhœa, although it is one of the most frequent symptoms, yet is not found at all stages of the disease, and that neither the enlargement of the follicles nor their ulceration necessarily produces the symptom." We have given both the data and conclusion of M. Chomel, so that each reader can judge for himself. Although diarrhœa is not necessarily produced by diseased follicles, yet it appears to us that the evidence is sufficiently strong to conclude that, when diarrhœa is present, it is the effect of this irritation: for medicine abounds with instances in which the same cause produces in different individuals different effects. If this question could be solved by arithmetical calculations, this inference would not be correct; but in this case our judgment is to be formed from probable not demonstrative evidence,—from evidence, in fact, admitting of degrees. The same change, as far as we have the means of judging, does not always produce the same symptom even in those diseases which fall immediately beneath our own cognizance, as affections of the eyes: indeed, even a constant lesion, as a tumour in the cranium, may produce intermittent symptoms, or none at all. We therefore require evidence of a more exact nature than the subject admits of, if no allowance is made for comparatively few exceptions. This is not a mere contention for words; for the great advantage of studying morbid anatomy is to endeavour to discover the connexion of the lesions with the symptoms or signs they produce.

M. Chomel in his general conclusion comes nearer to our views of the subject than in the passage previously quoted, but still we think he is too cautious in his inferences. He says, "the conclusion from all these facts is, that the various morbid changes of the follicles and mesenteric glands are not revealed to us by any particular phenomenon, and that all the symptoms, excepting perhaps diarrhœa, abdominal pain, and gurgling, are the expression of the influence of the disease on the whole system, of the disorders into which it throws the principal functions; and they are the effect of the disease itself, rather than of the lesion of the follicles."

M. Chomel next describes the *changes which are not constantly met with.* As our space will not permit us to dwell minutely on all, we shall only enter particularly on those concerning which there has been some difference of opinion.

*Stomach.* The colour of the mucous membrane is sometimes red or approaching blue, but, if there is no change in consistency or thickness, the colour cannot be regarded as denoting inflammation, unless symptoms of gastritis have preceded death. Symptoms of pain, &c. in the stomach are rare in the last days of typhus, when the patient is comatose. There was softening of the stomach in fourteen cases out of forty-two; in ten of these the mucous membrane of the great cul-de-sac was softened; in two the greater part, in one the whole, mucous coat; and in one all three coats. One in three appears at first sight too large a proportion to be accidental, but careful pathological researches have shown that this appearance is of little value; for that it is not more frequent in typhoid fever than in other fatal diseases. Thus, of twenty-four subjects who died of pneumonia in M. Chomel's wards, there was softening of the gastric mucous membrane in eight; in ten cases of peritonitis, three; in five who died of smallpox, two; and in other diseases in like proportion. The mucous membrane is rarely thickened: thinning often coincides with softening. In no case did M. Chomel meet with ulceration; M. Louis has in four instances.

*Intestines.* The alterations of colour are various. Generally the duodenum and jejunum are of a deeper colour than the rest of the small intestines. Deep redness often depends on position, as when there are zones of red separated by equal portions perfectly pale; or often when the lower part of the ileum alone is of a vivid red, from being thrust, by the distended stomach and large intestines, into the cavity of the pelvis. That redness often depends solely on position is seen where a few inches of the ileum near the cæcum are quite pale, whilst the rest is high coloured; the attachment of the cæcum preventing this portion from falling down: in this pale part the diseased follicles are most numerous. The redness is not greater around the diseased glands than at some distance. The colour of the mucous membrane of the large intestines is less changed. Softening of the mucous coat of the intestines is uncommon.

*Connexion of the Symptoms with the Changes in the Gastro-Intestinal Tube.* In only two cases out of fourteen, in which there was softening of the gastric mucous membrane, was there vomiting at an early stage, and in one other during the last few days. Of twenty-eight cases where no softening was found, there were five in which vomiting was a prominent symptom. Sensibility of the epigastrium was not greater where there was softening than where there was none. It does not seem that any change in the stomach during fever is marked by a prominent symptom; and a similar remark applies to the affection of the mucous membrane of the intestines.

Sanguineous infiltration of the mucous membrane of the intestines may be confounded with simple redness. The membrane is two or three times as thick as ordinary, having the shining and trembling aspect of a layer of black or red jelly spread over it. It is produced by sanguineous infiltration of the cells of the mucous membrane. Blood can be squeezed out by pressure with the handle of the knife, so as to give the membrane its natural appearance. It varies in extent from four inches to two or three feet; it is always continuous, never in zones, or in the most depending parts. It was found in seven out of forty-two cases: two of these had suffered from intestinal hemorrhage; a third had vomited blood; in two others blood was found in the small intestines; and in the remaining two it was, as in the other cases, easy to press out a sanguineous fluid. It is not certain that these two might not have had bloody stools, as in an hospital they might have been overlooked. There is an obvious connexion between this state and intestinal hemorrhages: of six cases where this symptom was present, there were four where this change was observed. It throws some light on the anatomical condition of the mucous membrane from which blood is poured, and refutes the common opinion that the blood flows from a vessel opened by ulceration. In four instances of death from other diseases, in which intestinal hemorrhage occurred, this appearance was found.

*Spleen.* Next to the intestinal follicles, the spleen is most frequently diseased. In almost every case where death takes place during the acute stage it is enlarged; but, with two exceptions, this was not the case in those who died early, or after the twentieth or twenty-fifth day. In ten cases out of forty-two it was softened, as well as enlarged, and in three almost fluid. In some instances it was harder and drier; generally when death took place after the thirtieth day. These changes cannot be connected with any particular symptom.

*Liver.* Sometimes softened: Louis observed this in about half his cases.

*Organs of Circulation, &c.* The blood is often deficient in fibrine. In thirty cases, in which the blood remaining in the heart and vessels was carefully examined, there were small fibrinous clots in six only; and in two of these acute inflammation had complicated the fever. Air has been found in the blood-vessels, particularly in the veins: in these cases which resemble thus far those that have been fatal from exposure to mephitic gases, the blood was in an advanced state of decomposition. Petechiæ and ecchymosis are observed during life in such cases, so that the decomposition of the blood probably commenced before death.

*Heart.* In seven cases out of thirty, there was softening of the heart, coincident generally with a similar state of other organs; in seven other cases out of the same number, the walls were flaccid. The softening was attended with paleness. Sometimes the inner membrane was a lively or deep red; in no case were there inflammatory depositions. The red condition often observed of the inner membrane of the aorta was probably owing to imbibition, as it was in proportion to the putrid state of the blood. Some experiments by Trousseau and Rigot strengthen this opinion: they discovered that a portion of a white and healthy aorta, steeped for a few minutes in the blood of some horses, particularly of those who had died of malignant diseases, was coloured of a vivid red: they also proved that, in living horses, the arteries inflamed with great difficulty, although irritated in various ways. The softening of the heart cannot be attributed to inflammation, as it coincided with loss of colour and with softening in the liver, spleen, &c.; all of which are not likely to have been inflamed at the same moment.

*Lungs.* Congestion of the posterior and inferior parts is frequent; for, as the vital strength declines, physical laws operate, and fluids accumulate in the depending parts. This was the case in eighteen instances out of forty-two, sometimes connected with softening: in eight cases there were marks of pneumonia, and in two pleuritic effusion.

*Brain.* Although its functions are most disturbed, yet it suffers fewest appreciable organic changes. Delirium, present in half the cases, is not explained by the pathological changes. Œdema of the meninges and bloody points are found, it is true; but these are met with as often in cases where there has been no affection of the cerebral functions as when they have been present, and also as frequently in other diseases as in this.

In thirty-eight carefully observed cases, there was

| | |
|---|---|
| Injection (venous) of the meninges in . . . | 4 |
| Œdema of the meninges . . . . . | 7 |
| General but slight softening in . . . | 6 |
| Serous effusion in the ventricles, (from a teaspoonful to a dessert-spoonful) . . . . . . | 12 |
| Bloody points . . . . . | 5 |
| Increased density . . . . . | 2 |
| Healthy state . . . . . | 15 |

*Emphysema.* Occasionally the body becomes emphysematous immediately after death.

The next division of the work before us embraces a subject concerning which much discordance of opinion has existed, the *Causes* of fever. The causes of diseases in general are sufficiently obscure, and the real amount of our knowledge of them is very small; yet few subjects are written about and discussed with greater prolixity or less doubt. The following table exhibits the exciting causes in 116 cases which fell under M. Chomel's observation, as far as could be ascertained by enquiry.

| | |
|---|---|
| 5 | patients attributed it to sudden cold when heated, |
| 6 | to deficient or bad food, |
| 4 | to mental depression, |
| 5 | to debility from other diseases, |
| 3 | to the action of a purgative taken for some indisposition, |
| 1 | to excess in drink, |
| 5 | to excessive fatigue, |
| 2 | to a violent physical shock, |
| 1 | to the effects of the sun, |
| 5 | were exposed to circumstances favorable to contagion, |
| 79 | no cause could be ascertained. |
| 116 | |

This table is much more satisfactory than the usual plan of enumerating the exciting causes of any disease, and it is the only method by which a writer can give his readers an opportunity of judging at once of the value of any statements. Many of our readers will feel surprised at seeing that no cause could be discovered for seventy-nine cases of fever out of 116; but M. Chomel's name guarantees the accuracy of the table, and his well-known diligence prevents our attributing the circumstance to any want of care in the examination.

Other tables follow, in which no cause could be discovered in nearly half of a large number who were affected with pneumonia. We can hardly believe that this is possible; and yet the same proportion of cases have most probably presented themselves to our own notice, in which no cause of disease could be ascertained, which have impressed our attention less strongly than they should do; owing perhaps to one of the defects in our mental constitution, by which positive evidence makes so much greater an impression than negative; the frequent absence of any circumstance striking us with much less force than its occasional presence.

The tables showing the exciting causes in pneumonia are well worth comparing with the one given above. Out of 137 cases, twenty-eight attributed the attack to exposure to cold; a small proportion, indeed, if we judge by our own traditional prejudices in favour of this agent, but a much higher number than any we shall find in the column of the previous table. In pneumonia, the preceding operation of cold is too frequent for us to consider it as a mere coincidence; but in fever we cannot draw the same inference: twenty-four who were attacked with pneumonia were debilitated by previous catarrh; whereas fever, with rare exceptions, attacks those enjoying perfect health: pneumonia attacks all indiscriminately, not so fever: twenty cases of pneumonia were second attacks, whereas, in 130 instances of fever, the individuals had never suffered previously. These comparisons show that there are marked differences between fever and inflammation.

The question as to the age of those most subject to fever is examined in the same tabular way. It is most frequently met with from eighteen to thirty, when the bodily powers are greatest; it is rarely observed after forty, and perhaps no case is on record where the patient was more than fifty-five. From those under fifteen being sent to the hospital for children, these details do not embrace the young. M. Chomel, however, has no fear that he shall deceive, when he says that the frequency diminishes until ten, beneath which age it is very rarely met with. Another circumstance peculiar to the typhoid fever which M. Chomel describes, is that more than two-thirds of the whole number of patients had lived in Paris less than two years, and only two were born in Paris. It appears to be necessary to be habituated to the climate to resist the causes, whatever they may be, of this affection.

The question of *Contagion* is next examined. The majority in France are anti-contagionists, in England the other way. The direct evidence in favour of contagion, in the table of causes previously given, is very slight; when compared with the proofs of contagion, apparently of the most convincing character, brought forward by Dr. Tweedie, of the London Fever Hospital; Dr. Marsh, of Dublin; Dr. Alison, of Edinburgh; and Dr. Millard, of Glasgow; each of whom had considerable experience on an extensive scale. M. Chomel impartially states their evidence, but adds that sufficient anatomical descriptions of the state of the intestines cannot be obtained, to prove satisfactorily that the typhoid fever of Paris and the typhous fever of London, Dublin, and Edinburgh, are one and the same disease. He concludes a very clear and fair chapter by three propositions:

1. That the general opinion of French physicians that the typhoid fever is not contagious, is not proved.

2. That, if it is contagious, it is only so in a feeble degree, and with the concurrence of circumstances at present undetermined.

3. That, if ulterior observations prove the identity of the anatomical lesions in typhous and in typhoid fever, the identity of the two affections will be proved, as well as the question of contagion.

M. Chomel alludes (as he could not fail to do,) to the little attention paid in this country to the state of the follicles of the intestines.

*Varieties of Typhoid Fever.* The sketch of fever which has been given embraces all the symptoms, but in no one case do they all meet; some symptoms excluding others, or being constantly united. The concurrence of particular symptoms constitutes varieties of fever, to which distinct names have been given by authors, as if they were distinct affections.

1. *Inflammatory* Typhoid Fever. This is frequent, particularly in winter: those of a sanguine temperament, and from twenty to thirty years old, and subject to hemorrhages, are liable to it. When well marked, the peculiar symptoms occur early; such as fulness and frequency of the pulse, hot skin, dryness of the throat, thirst, loss of appetite, oppression, and other general symptoms common to inflammatory affections; but, besides these, there are constant headach, muscular debility, disposition to hemorrhages, dry tongue, diarrhœa, typhoid and miliary eruptions. The form changes generally to the adynamic and ataxic about the seventh or eighth day, sometimes earlier. In two cases only out of forty-two fatal ones did the inflammatory form continue throughout the disease, and in one of these cases death was produced by perforation of the bowels. During five years, during which these cases were collected, M. Chomel saw no other instances of inflammatory fever which were fatal, and he has never met with inflammatory fever which was not a variety of the typhoid affection.

2. *Bilious* Typhoid Fever. Most frequent in summer and autumn. In two cases out of forty-two fatal ones, there were bilious symptoms at first, giving way to more serious ones. Five others were cured where these symptoms continued throughout. The symptoms are—yellow skin, especially around the lips and alæ nasi; frequent nausea, and vomiting of bile; bilious stools; bitterness and dryness of the mouth; yellow or greenish coating to the tongue; tinnitus aurium; depravation of taste, smell, and touch. The duration of these symptoms is seldom beyond the seventh to the fifteenth day.

3. *Mucous* Typhoid Fever. This, like the bilious, seems to depend much on localities: it is seldom well marked in Paris. The symptoms are—great debility; pale or swollen face; muscles soft; mouth pasty; breath, saliva, perspiration, and urine, of an acid odour; stools mucous or glairy: after a short period it is replaced by the adynamic or the ataxic form. Two out of forty-two fatal cases had these symptoms.

4. *Ataxic* Typhoid Fever. One of the best marked, most frequent, and most generally fatal forms. Ten out of forty-two were ataxic: four of these were unmixed throughout, and death ensued on the eighth, ninth, and twelfth days; two were preceded by the inflammatory, and two by the adynamic symptoms. This variety is distinguished by a remarkable disturbance of the functions of relation; as delirium, cries, threats, efforts to strike or escape; sometimes by mild delirium, heaviness, alteration or perversion of the senses, twitching of the tendons, convulsions, rigidity, &c. In other cases there is a remarkable discordance between the symptoms: thus, whilst the pulse is rapid, the skin is not hot, or one part is cold whilst the rest is very warm; or, whilst the face expresses a disease almost inevitably mortal, the pulse is hardly affected. Frequently the delirium is not in proportion to the other symptoms, either less or greater. Sometimes a sudden improvement leads the practitioner to doubt his diagnosis: the benefit is, however, temporary only. In some cases the patient is perfectly restored to his senses before death. Ataxic symptoms do not belong exclusively to fever, but may coexist with visceral inflammation, puerperal, eruptive, and other acute diseases.

5. *Slow Nervous* Typhoid Fever. The symptoms are—a general indifference, great lassitude, heaviness, dejection; slight headach; pulse frequent and weak; constant wakefulness; no thirst, although the mouth is dry; if there is delirium, it is not violent, and consists of a confusion between thought and action; the patient mutters: in unfavorable cases the strength diminishes, and the stupor increases, with other adynamic symptoms; in favorable ones, the patient gradually throws off the drowsiness, or suddenly, as if awaking from sleep.

6. *Adynamic* Typhoid Fever. The most frequent form, adynamia being marked in twenty-six out of forty-two fatal cases: in ten of these, adynamic symptoms were present throughout, and in sixteen at the termination only. The predominant symptom is muscular debility, which may gradually simulate paralysis. These patients, with every appearance of strength, can neither lie down nor rise up in their beds without help, or even turn on one side. Towards the termination they lie immoveable, and after many hours are found in precisely the same position in which they had been left. There is commonly great mental debility, commencing with early stupor. In bad cases, or at an advanced period, the patient does not answer questions which are put to him, and his unmoved features show that he has not understood them: after a loud question he may direct his eyes momentarily towards the speaker. Headach diminishes as adynamia increases, and is replaced by wakefulness, or constant unquiet dreams. The mouth is covered with a thick layer of dry mucus; great meteorism; often no sensibility on pressure; stools generally fœtid and involuntary; sloughing of the parts pressed upon; urine and sweat fœtid; petechiæ; skin at first warm and dry, afterwards cold; pulse feeble, trembling, at first rapid, latterly slower. This state sometimes lasts long.

Such is the division of the varieties of typhoid fever which M. Chomel approves: he judiciously adds, that they will commonly be less defined, and often running into one another. This caution is applicable to all nosological divisions, for they are the work of man, not of nature; and, if this is not kept in view, the student in particular will be constantly deceived and led astray. The principle on which divisions of fever are made is of importance, as our judgment is liable to be influenced by mere names, and for this reason there are difficulties which beset all classifications. The school of pathological anatomists, of which M. Chomel is one of the most ardent disciples, has improved our knowledge of fever, by leading our attention to the state of the particular organs, which they have shown are subject to inflammation, arising during the course of the disease; whereas the older writers* directed their attention almost exclusively to the general condition of the patient. Both the general state and the intercurrent inflammations are important grounds for divisions, and neither can with propriety be exclusively adopted. The division which Cullen adopted of synochus, synocha, and typhus, obscured the subject by directing the attention to the general condition of the patient, to the neglect of local complications. On the other hand, divisions founded on the local complications only would be defective, as they refer in no way to the peculiar character of the fever itself. Dr. Southwood Smith has endeavoured, in his work on Fever, to found his divisions on both these principles, and, although his classification is rather too complex, its principle is correct. M. Chomel has adopted the exclusive principle on which Cullen based his system; and, for the reasons just stated, we do not think it is the one which is likely to be the best guide to treatment; the great object of our art. We shall make some further remarks on this subject when we arrive at its practical application.

*Diagnosis.* This is sometimes extremely difficult. It is prudent not to give a decided opinion during the first three or four days; for, when the symptoms are not very decidedly marked, they differ little from the precursory fever of many eruptive diseases, as small-pox, scarlatina, measles, of some catarrhal affections, or latent visceral inflammations. The long duration of the febrile condition is an important characteristic. Whenever febrile symptoms, which cannot be referred to any appreciable lesion, last eight or ten days, there are strong grounds to presume that the glands of Peyer are diseased, and when, on the other hand, a febrile disease, of the nature of which we were doubtful, terminates in a few days, it is not this affection. Between the sixth and twelfth days, symptoms which clear up the diagnosis generally appear, such as meteorism, typhoid

* Sydenham was an exception.

eruption, stupor, epistaxis, hemorrhage from the bowels. At a later period still, there is less difficulty; for, even if the symptoms during the first and second periods have been absent, those which belong to the third remove all doubts: these are intestinal hemorrhages, sloughing, involuntary stools, and other marks of adynamia. Enteritis is one of the diseases most likely to be confounded with it; but in enteritis the fever is less, and the stools are more numerous and painful, the diarrhœa lasting during the whole disease. In some cases of fever, diarrhœa is a late symptom or is absent. The prostration of strength is much less, and adynamic symptoms, as stupor, delirium, involuntary stools, as well as the eruption, meteorism, and sloughing, are rare. It may last many months without producing that debility which typhoid fever does in a few days. When a patient is first seen in the adynamic state, and no history of the case can be obtained, the diagnosis becomes difficult. There are many diseases, even then, with which it cannot be mistaken. The acute visceral inflammations of old men, for instance, speedily put on an adynamic character, but their age prevents the suspicion of typhoid fever. Phlebitis and partially retained placenta may simulate this affection, but the local causes of the disease prevent error. It is rare also in the puerperal state. In some cases the symptoms throughout are so mild as to leave us in doubt. When a disease is prolonged to the fifteenth day, and the only symptoms have been loss of appetite, malaise, greater or less fever, some liquid stools, without any marked change in the muscular contractility, we must regard it as typhoid fever; no other disease follows a similar course. Experience shows that from the fifteenth to the twentieth day, or later, some well-marked symptoms may occur, and, if death has accidentally taken place, the characteristic lesions have been discovered. The progress of the disease, more than the actual symptoms, distinguishes some ataxic cases from cerebral inflammation.

*Prognosis.* Few diseases are so fatal. Out of 147 cases in the clinical wards of the Hôtel Dieu, between 1828 and 1832, forty-seven died, or one in three. Though a mortality of one in three is a very large proportion, any inferences unfavorable to the treatment of fever should for many reasons be made with caution and charity. The mode in which patients are distributed to the various hospitals in Paris, is brought forward as one excuse for such fatality. All the hospitals being under the direction of government, a central board of medical men is appointed to examine the patients who apply for relief, and to distribute them among the different hospitals. This board meets near the Hôtel Dieu, so that the severest cases of fever are often sent there, as it is the nearest place. M. Chomel is also the professor of clinical medicine, and the most serious cases are sent to the clinical wards. These reasons would account for a greater apparent mortality than under other circumstances, if we did not find that during several years, whilst M.

Chomel was physician to La Charité, the mortality in about the same number of cases was rather greater. M. Louis founded his "Recherches sur la Gastro-entérite" on 138 cases of fever treated by M. Chomel, and out of these there were fifty deaths.* The average of one in three seems to be therefore independent of these local causes. In order however to come to any fair conclusion, we should have the means of comparing these results with others in the same city, founded on an equal number of cases treated in a different manner. Particular modes of treatment are often brought forward as useful, and the proportion of successful cases seems to justify the means recommended, but a more extensive series of experiments with the same remedies overthrow all the previous conclusions. For instance, in a subsequent part of this volume, M. Chomel gives tables of the mortality during his experiments in treating fever with the chlorides: in 1831-32, when the first trial was made, the ratio of deaths was one in ten, or it might be said one in nineteen; whereas in the next two years it was a third! Again, no comparison between the mortality in the typhoid fever in Paris and that in this country could be safely drawn, as there are no means of estimating the degree of severity of the prevailing epidemics in the two countries. If it were not so, the returns furnished by our fever hospitals would be decisive against M. Chomel. But, after making all these allowances, we still must conclude that no benefit could have been derived from any mode of treatment, where the mortality was so high, and so permanently high, for a long series of years, equal indeed to that of malignant cholera itself. As we feel ourselves justified in this country in attributing success in dangerous cases to the remedies employed, which are much more decided, we may safely strengthen our criticisms on M. Chomel's treatment by a reference to this high ratio of mortality. This we shall reserve for its proper place. We shall not dwell on the details of M. Chomel's tables illustrative of the prognosis, but only give the additional and more correct information which he has brought forward. Without questioning the utility of weighing each symptom to discover its value in prognosis, yet we would put the younger of our readers on their guard against relying too much on this kind of information. It is not one symptom on which any correct decision can be formed, but on the case as a whole, and experience alone, that is, attentive observation and reflection upon numerous cases, can give the practitioner any feeling of certainty as to his decision of the termination of a doubtful case. Constant errors are made in judging of the probable issue of diseases, and particularly by those who have had limited experience: the power of distinguishing diseases is much more quickly acquired.

Fever is less dangerous in patients under eighteen years of age, and more dangerous after the age of forty. No appreciable diffe-

* Recherches sur la gastro-entérite, &c. par P. Louis. Vol. 1. p. ix.

rence is observed in regard to sex. Previous feebleness of the system does not appear to act unfavorably. Two out of four patients who attributed fever to moral causes of depression died. Of sixteen patients who admitted that they had taken stimulating drinks at the commencement of the attack, three only died. M. Chomel concludes that those cases are most dangerous where the attack was sudden. The tables given, however, indicate the opposite, the mortality being rather less than one in three where the attack was sudden, and slightly above one in two where there were premonitory symptoms. (P. 433.) There is probably some numerical error. If during fever there is a decided remission, followed by an aggravation of the symptoms, the termination is generally fatal. There is less danger when the form of the disease does not change: the ataxic is in such cases the most fatal. Complicated cases are very fatal: thus, of thirteen cases of inflammatory adynamia, eight died. Many symptoms, when they become intense, are important in the prognosis. When delirium is early and violent, it is very unfavorable. Of forty-two fatal cases, twenty-two were violently delirious. When it consists in a dreaming state from which the patient can be roused, there is less danger. Of eighty patients who recovered, twelve had this mild delirium. Involuntary evacuations, when passed without consciousness, constitute a bad sign. Of thirty cases, in which this symptom was present, thirteen died. Constant and general twitching of the tendons is highly unfavorable. In five cases with general convulsions death was speedy. Coma is one of the most fatal symptoms; it should be distinguished from stupor, in which the patient's attention can be roused. Of seven patients with intestinal hemorrhage six died. M. Chomel does not think deafness unfavorable. The expression of the face is important: when emaciated and shrunk, (facies Hippocratica,) death is at hand; whilst improvement in intelligence of expression is often the first sign of amendment. If the pulse exceeds 120 or 130 it is bad, when 150 or 160 death is near. When it becomes slow after having been rapid, without symptoms of improvement, it is a fatal symptom, unless proper means to relieve the patient are not employed. Perforation of the intestines, and erysipelas of the face, are generally fatal complications. The danger of inflammation of the lungs is in proportion to its extent and to the general condition of the patient. When it occupies a considerable portion, or the whole of one lobe, and is not arrested, it is fatal, even before it passes into the second and third stage. Circumscribed pneumonia is often discovered in those who have extensive suppurations on the sacrum, and is dangerous. As pneumonia is often latent, considerable attention should be paid to the lungs. In three patients inflammation of the larynx and epiglottis took place, and was fatal. The injurious effects of sloughs on the sacrum, heels, &c. have been exaggerated. In seven cases, only three died, and in those which recovered the extent of the ulcers was truly alarming. Abscess in the external parts was observed in six, all of which recovered. They were not found in parts subjected to pressure.

*Treatment.* M. Chomel employs the rational mode of treatment, in which the disease is treated according to the symptoms which may be present, and not according to any uniform plan. By this mode, none of the specific modes of cure is excluded, though none is exclusively adopted. The antiphlogistic, the antiseptic, the tonic plans are not individually adhered to in every case, but are applied according to the form which the fever may assume. This is called rational treatment, as it supposes that the practitioner reasons on every case; it is also called symptomatic, from the attention which is necessarily paid to symptoms.

In the simple uncomplicated forms, M. Chomel prescribes refreshing drinks, such as lemonade, orangeade, solution of syrup of currants, pure water taken at short intervals, emollient fomentations and poultices to the abdomen, if it is painful; washing the body with vinegar and water, or simple baths, if there is much heat; mucilaginous lavements repeated many times daily; cold compresses to the forehead, if there is much headach, and warm or mustard poultices, if there is any tendency to drowsiness or forgetfulness. He also commences by taking some blood from the arm, as he agrees with M. Louis that this has a favorable influence on the duration of the disease. If the headach is intense, or if there is much abdominal pain, leeches may be applied behind the ears or to the anus. If the stools are scanty, mild laxatives, such as whey with tamarinds, neutral salts, &c. If there is diarrhœa, it should be restrained by mucilaginous drinks, gum or rice water, small lavements of starch. Free air and absolute cleanliness are indispensable: great care should be taken that the urine and fæces passed involuntarily should be immediately removed. When amendment commences, the emollient drinks may be exchanged for aromatics and gentle bitters: diet improved, such as vegetable jellies, weak broth, wine and water, &c. When the symptoms are more urgent, this expectant treatment is replaced by a more vigorous one.

*Treatment of Inflammatory Typhoid Fever.* This requires the antiphlogistic treatment according to the age and strength, but by no means with the same vigour as in simple inflammations; for it must be remembered that adynamic symptoms frequently follow inflammatory; there is therefore a necessity of husbanding the powers of the patient. Another reason for the same caution is that inflammation frequently springs up in the most debilitated subjects. Therefore, after taking blood once or twice, generally and locally by leeches, these means must be laid aside, and complete abstinence, with the remedies just mentioned, trusted to. The only cases where general bleeding is indicated in the second and third periods, would be when inflammation attacks patients who are not greatly debilitated. Great caution is required in all such cases.

*Treatment of Bilious Typhoid Fever.* The bitter taste in the mouth, great thirst, &c. cause the patient to request cooling drinks, ripe fruits, &c., which should be allowed. M. C. has not found emetics and purgatives so useful, nor bleeding so dangerous, as the physicians of the last century state. Emetics may be used at the commencement of a sudden attack, if the stomach appears to be loaded, but cooling drinks and fruit generally relieve the bad taste in the mouth.

*Mucous Typhoid Fever.* This is treated like the simple, except that acid drinks are given instead of emollients, and slightly bitter and aromatic infusions of indigenous plants, such as are made no use of in this country except by the poor, and therefore not at all equivalent to our pharmaceutical bitters and aromatics.

*Treatment of Ataxic Typhoid Fever.* The treatment of this variety is very difficult: the antiphlogistic, tonic, and antispasmodic plans have all had their exclusive supporters. The treatment however must vary. If inflammatory symptoms are present, the antiphlogistic treatment, and if the adynamic, tonics must be recommended. When there is no precise indication, the expectant treatment is to be followed.

*Treatment of Adynamic Typhoid Fever.* When there is stupor, unusual prostration of strength, weakness of the pulse, faintness in the sitting posture, and involuntary passing of stools and urine, we must use bitters and aromatics, such as bark, chamomile, and sage in draughts, lavements, baths, and external applications; with wine, camphor, and ether: if the symptoms increase, the doses must be larger, and the wines of Spain given instead of those of France. Extract of bark, by the mouth and in lavements, in doses of one to two ounces a day, is given by M. Chomel in preference to quinine, if the stomach will bear it, as he doubts whether the sulphate of quinine contains all the tonic powers of bark equally with its febrifuge and antiperiodic principles. In this state tonics and excitants, instead of aggravating the lesions of the intestines, exercise a favorable effect upon them. The intestinal ulcers are analogous to cutaneous ulcers in similar subjects, which are improved by stimulating applications. In three instances where the patients died during the tonic treatment, the ulcers in the intestines were evidently cicatrizing. The tonic treatment was followed in nine patients, all of whom when it was commenced were in an alarming state of prostration, and six of these recovered. It is important that tonics should be given before the strength is too much exhausted, and yet not during reaction. The exact time must be determined at the bed-side, as no exact rules can be laid down. If delirium or other signs of cerebral congestion exist, wine should not be given, as it almost inevitably aggravates the symptoms. M. Chomel commonly gives wine in spoonfuls, at first once or many times daily, increasing the quantity as debility increases. The lighter wines he gives with other drinks, in the proportion of a fourth, a third, or half; the stronger wines pure. In some cases the benefit is immediate: the pulse rises, the heat of the skin increases, and the expression improves. Ether is particularly useful when it is necessary to raise the powers rapidly, but its action is transient; it should be given with bark. Camphor is only employed by M. C. in lavements with bark, when debility is great. Bark in infusion, decoction, or still better only macerated in water, and sweetened with syrup of lemon, is one of the best drinks. Also infusions of serpentaria, cascarilla, and sage. The tonic treatment is rarely necessary in the first stage, and should never be tried then except with great reserve. In the second and third stages we may employ it with more confidence and energy. Several excellent cases are detailed in which success followed this treatment in apparently hopeless cases. M. Chomel mentions the application of revulsives and of warm and cold baths, but states nothing decidedly as to his own opinion of their efficacy.

*Treatment of Particular Symptoms and Complications.* Hemorrhages are rarely so profuse as to require special treatment. Epistaxis may render plugging the nostrils necessary, and if the discharge of blood from the bowels is great, cold or iced water in draughts, lavements, and external applications, extract of rhatany, &c. should be tried. Great care should be taken to prevent the formation of sloughs: when the fever has lasted any time the parts pressed on should be examined, and if there is that redness over the sacrum which precedes sloughing, the patient should be so supported as to lie on the side or even on the belly. When the eschar has formed, it should be covered with diachylon plaster; when it has fallen, the wound should be dressed as an ordinary ulcer. M. C. has not tried Dr. Arnott's water bed. The treatment of local inflammations attacking a debilitated subject is very difficult. Local bleeding, particularly cupping, must be cautiously employed, if the strength will permit. But generally the adynamic condition forbids it, and the tonic treatment must be pursued, whilst the local disease is combated with epispastics, as blisters and rubefacient plasters. In erysipelas of the face, the blood should be directed towards the feet by sinapisms, or very hot flannels covered with oiled silk. All the cases of perforation of the intestines which have fallen under M. Chomel's immediate observation have been fatal. Perfect rest and abstinence was the treatment adopted, but if other cases should occur he proposes to try the plan suggested by Dr. Graves of Dublin, and put into execution by himself and Dr. Stokes, of giving large and repeated doses of opium, so as to preserve the intestines in a complete state of rest, in order to prevent the further escape of fæcal matter into the peritoneum, and to allow nature to close the opening by adhesive inflammation. Opium is admirably calculated to fulfil this intention, by putting a stop to or weakening the peristaltic action of the bowels, and by soothing the excessive pain. These accomplished physicians have had some cases to justify the utility of the practice, and although it has not often succeeded, yet it has never wholly failed to assist nature under this distressing accident.' We would refer those who desire complete information on this important subject to the original paper of M. Louis on perforation of the intestine in his "Mémoires ou Recherches Anatomico-pathologiques," p. 136 et seq.; to the 5th vol. of the Dublin Hospital Reports; or to an able article, embracing both pathology and treatment, by Dr. Stokes, in the Cyclopædia of Practical Medicine, (art. *Peritonitis.*) The state of the intestinal tube will explain the frequency of tedious convalescence, and the accidents to which those are subject who are recovering from this disease. When the heat of the body diminishes, even although the frequency of the pulse continues, some liquid food may be given, such as veal and chicken broth, "le lait de poule," milk and water, &c. augmented gradually until solid food can be digested. If the appetite does not return, and the patients are very weak, bitters should be given. Country air is very favorable to convalescence.

Such is a sketch of M. Chomel's mode of treatment. The objection which we made to his adhering exclusively to the old division of varieties, which was based on the general condition of the patient, from its tendency to direct the attention entirely to this condition, to the neglect of the state of the various organs, is borne out by these directions for treatment, in which local complications are almost entirely overlooked. The minute attention which M. Chomel had given in the previous chapters to the various organic changes in fever appears to be of little or no use in his practice, as the practical rules relate almost wholly to the general state of the system on which his varieties depend, and which he admitted could not be referred to any local cause. The application of the information derived from morbid anatomy is made to the diagnosis and prognosis, but does not extend to the treatment. There are undoubtedly two opposite states of the system to which the terms inflammatory and adynamic may be applied, which appear to exist independently of local disease, and to depend greatly on the "constitution" (whatever that may be) of the prevailing epidemic; and practitioners should be well aware of those states, and keep them in view in the treatment, but the due recognition of complications of fevers from inflammations of various organs is of vast importance. Visceral inflammation may exist with either of these opposite general conditions, and the success of any treatment must depend on the detection of the local disease, and the modification of the means made use of, according to the general state of the patient. M. Chomel, we think, has not applied his knowledge of morbid anatomy where it would be both applicable and beneficial. He has stopped short too soon; he has not carried out his principles as far as they will legitimately go. With other modern pathologists, he has confirmed, by dissection, the views of Sydenham, as to the frequency of local complications of fever, derived from an examination of symptoms alone, but he has not followed to the full extent the same example, by making the information thus acquired subservient to the great object of our art, the cure of disease.

There can be no doubt, from these directions for treatment, that M. Chomel leaves much more to nature than we do in this country. The active antiphlogistic treatment is confined to bleeding and leeching; and the cautions against producing adynamia, and the directions to husband the strength, indicate that M. Chomel regards depletion as an "anceps remedium."

That cautions are not necessary against depleting too largely in fever, even when inflammation is present, we by no means wish to assert; but it must be borne in mind that these cautions come from Paris, where what is called an English bleeding is hardly considered justifiable in the most acute inflammations of vital organs.

If one or two small bleedings and leeches fail to remove inflammation, M. Chomel has no other remedies than complete abstinence, emollient lavements, diluents. In this country we should imagine such directions meant that, if bleeding failed, the case was altogether hopeless, as far as our remedies were concerned; and in the majority of cases this would probably be the truth. Those powerful antiphlogistics, purgatives, are not mentioned, but in their stead emollient lavements.

Purgatives appear to be banished, from a fear that they may increase the irritation of the follicles of the intestines; a fear which has sprung from too exclusive devotion to morbid anatomy. That active purgatives, particularly in the early stages of fever, will increase the follicular irritation, is a completely theoretical objection. The reasoning on which it is founded will not bear examination, and our experience in this country experimentally contradicts it. M. Chomel has remarked with some surprise that he has found ulcers of the intestines cicatrizing in patients who died whilst under a course of tonics and stimulants, instead of being aggravated by them: such a remark could have been only owing to his imagining à priori that the passage of stimulants over ulcers in the intestinal canal must injuriously irritate them. We venture to hope that M. Chomel would feel equally surprised and pleased with the effect, in many cases, of purgatives, although their exhibition would at first be opposed to his notions of their effects on the mucous membrane. It is pleasing to see, by some of the recent French periodicals, that MM. Andral, Guersent, Baudelocque, and other physicians attached to hospitals, are conquering their prejudices against purgatives, and trying them in fever: the results we shall look forward to with curiosity and interest. Another remedy of great power, particularly in those cases of inflammatory complication where depletion cannot be safely persevered in, is calomel, given in small and repeated doses, especially when combined with opium, so as to affect the system. This is not alluded to by M. Chomel, and its efficacy does not appear to be recognized or even tested in Paris. The employment also of the "cold dash," or pouring cold water from a jug over the head, where there is cerebral inflammation, is overlooked. It is a remedy of extreme value: in the ataxic variety of typhus with furious delirium, we have seen it almost instantly produce tranquillity. M. Chomel is most vigorous in his tonic and stimulant treatment of the adynamic stage, and some of the cases which he has appended are instances of success even under the most disadvantageous circumstances. The water-bed of Dr. Arnott is particularly adapted to cases of fever where there is extensive sloughing: the only complaint which patients make to it, that they cannot alter their position without great difficulty, would not apply to these cases, where voluntary motion of any sort is frequently impossible under any circumstances, and where it is advantageous that the patient should remain in the position in which he was placed.

The two chapters which conclude this volume are added by M. Chomel himself. The first contains an exposition of the treatment of fever with the chlorides, and a careful estimate of the results. M. Chomel, although evidently a reader of the medical literature of this country, does not seem to be aware that Dr. R. Reid, of Dublin, had, so far back as 1826, made experiments with these medicines in Fever, and published a paper on the subject in the Transactions of the King and Queen's College of Physicians for 1827. M. Chomel states that he was induced to try them, from a hint given him by a young physician frequenting his wards, and from the little efficacy of any known method of cure. He chose the chloride of soda, and exhibited it, not instead of other remedies, but in addition to them. One grain to a grain and a half was dissolved in each ounce of sweetened gum water, or slightly bitter infusion, if the first produced nausea; and the patients were directed to drink as much of this as they possibly could. The greater number took from three to five cupfuls, each holding eighteen ounces. Mucilaginous lavements, containing the same proportion, were ordered night and morning, with lotions four times daily over the whole body of pure chloride of soda. The poultices covering the abdomen were sprinkled with it; a pint was mixed in each bath; and the bedclothes, furniture, and vessels were sprinkled with it many times a day. It was tried in those cases only where there was no doubt as to the diagnosis, where the fever was of a dangerous character; and in the first or commencement of the second stage, as at a later period nothing could be concluded.

During the summer of 1831, five cases were thus treated. The symptoms of the first two were so aggravated, that M. Chomel resolved to wait their termination before he repeated the experiment on others, and, even after six or seven days, there was in one patient so great a prostration of strength, that the chloride was replaced by powerful tonics. Since this time he has often associated them. This patient, as well as the other four, recovered. Of fifty-seven patients treated in the common way sixteen had died, or about one in three, [one in three and a half.] From November 1831 to August 1832, twenty-three patients were admitted: fifteen were treated with chlorides, and eight otherwise: five of these eight were mild cases, and recovered; of the three fatal ones, two died shortly after admission, and there was some doubt as to the diagnosis of the other. Of the fifteen others treated with chlorides, two only died; and, in one of these, besides the peculiar lesions, there was partial hepatization of both lungs and tubercles. To prove that the thirteen cases which recovered were severe ones, and not selected, the following table of the principal symptoms in each is given.

1st patient. Tongue dry; meteorism; bloody stools.

2d. Extreme prostration; deafness; involuntary stools. Bark.

3d. Delirium.

4th. Delirium; meteorism; involuntary stools.

5th. Fuliginous mouth; considerable physical agitation.

6th. Involuntary stools; trembling of the lower jaw.

7th. Bilious vomiting.

9th. Meteorism; involuntary discharge of stools and urine.

10th. Fuliginous mouth; involuntary discharge of stools and urine.

11th. Delirium; disordered movements; fuliginous mouth; stools and urine discharged involuntarily.

12th. Meteorism; involuntary discharge of urine.

13th. Meteorism; dry mouth; considerable stupor.

If the results of both years are united, it appears that, of twenty cases treated by chlorides, only two have died, and that the death of one of these was owing to double pneumonia and tubercles; so that it might be said, that there were eighteen successful cases to one unsuccessful. M. Chomel, however, considered the number of cases too few to deduce from them any general propositions as to the efficacy of the treatment; and, indeed, during the next fifteen months, the results were far less satisfactory. From November 1832 to March 1834, (when the work went to the press,) fifty patients with typhoid fever were admitted; thirty-seven of whom were thus treated, and thirteen by the usual method. Of the thirteen, there were eight whose symptoms were so mild that the diagnosis was formed tardily or imperfectly: in three others there were complications, (particularly pneumonia,) which prevented the use of the chlorides: two were brought at a very advanced stage: five of the thirteen died. Out of the thirty-seven treated with chlorides, twelve died, twenty-five recovered. Of the latter, four or five were mild cases, and fourteen very severe. Of the twelve who died, one was convalescent when he was cut off by cholera, and, on examination, ulcers of the intestines almost completely healed were found; a second was, when convalescent, attacked with fatal pneumonia; a third, during convalescence, died from perforation of the lung from tubercles; a fourth was brought into the hospital in an unconscious state, almost dying, and lived but few days; two others had double pneumonia. Consequently, if from the large number of twelve deaths in thirty-seven cases, are deducted three individuals who died, not of typhoid fever, but after its termination, of cholera, pneumonia, and perforation of the lungs, and if these three cases are added to the twenty-five who were cured, and if from the nine other cases are deducted one who took the chlorides only two days, and the two individuals affected with double pneumonia, an almost constantly fatal disease, (more dangerous consequently than typhoid fever,) and therefore having a greater share than the latter in the extinction of life, the number of deaths will be reduced to six, and that of cures be twenty-eight.

From a general view of these details, it appears that, from the summer of 1831 to March 1834, fifty-seven patients have been treated with chloride of soda of whom forty-one (forty-three?) have left the hospital cured;
sixteen (fourteen?) have died; that if three who died of other diseases (cholera, pneumonia, and perforation,) after the termination of typhoid fever, are added to the forty-one (forty-three?), and if these three, as well as four others, the particulars of whose cases have been before mentioned, are deducted from the number of deaths, there will be a mortality of nine (seven?) in fifty-three, or nearly one in six (seven and a half?); whilst the mean mortality of cases of typhoid fever treated by the usual method in M. Chomel's wards at La Charitè and l'Hôtel Dieu, is one in three. Thus, at La Charitè, from 1822 to 1827, of 138 cases, fifty died; in 1827-28, five of eighteen; at the Hôtel Dieu, in 1831-32, sixteen of fifty-one; in all seventy-one deaths out of 207 cases, or a little more than one-third.

If the results of the treatment with chloride of soda are compared with these, the difference is very great; for if those cases in which the patients died from causes independent of fever are deducted, the mortality is only one in six (seven and a half?); or, if only those three cases are deducted where death ensued after the termination of the fever, from accidental causes, and those included where in one case the chlorides had only been taken thirty-six hours before death, another who had tubercles, and two others with double pneumonia, there will still be thirteen (eleven?) deaths in fifty-seven cases, or one in about four and a half (five and a half?).

Some error has crept into M. Chomel's calculations, as his *resumé* differs considerably from the previous numerical details, so that he under-rates considerably the benefit of the treatment he proposes. By the minute details, the proportion of deaths is only one in seven and a half, (deducting the seven cases;) whereas, M. Chomel states it as only one in six. We have placed in the resumé, with a query, the figures calculated according to the previous details which are accurately copied from the original; the error is of considerable importance, and requires rectification, one way or the other, by the author.

We have given M. Chomel's data, in order that all may judge as to his inferences. There is a source of fallacy in his calculations, the due importance of which should be estimated. A comparison is made between two sets of cases, differing in this essential particular, that a selection is made in the one and not in the other. If we take the whole number of cases treated with chloride of lime, which are fifty-seven, we find that forty-three are cured and fourteen died, which is one in three and a third; whilst the average of a much larger number of cases treated in the ordinary way is rather above one in three: the approximation then is singularly near. But M. Chomel deducts three cases, in which the deaths can be explained by cholera, perforation of the lung, and pneumonia, during convalescence, and adds them to the cures; by which he diminishes the mortality to one in five and a half. In order to compare this with the general table, we should be satisfied that a similar deduction would be made in that for such accidental deaths. The case of cholera might have been, under all circumstances, deducted; but is it probable that the other two, which were from diseases the sequelæ of fever, would not have been placed under the general head of deaths from fever? The four other cases, the deduction of which diminishes the mortality still more, were those where life was destroyed by inflammation of a vital organ or who lived a very short time after they were brought into the wards. Now, on examining those cases mentioned in the preceding calculations of fever under ordinary treatment, nearly all the deaths appear to have taken place under these two circumstances: twenty-one cases of fever were treated in the usual manner; thirteen recovered, and eight died; a mortality of nearly two thirds: but of these eight, three died of pneumonia, and four were brought in at a very advanced period.

M. Chomel has evidently selected the cases most fairly to test his new treatment, neither taking the very mild ones, nor the very neglected; but still a selection was made in the one case and not in the other, and this alone interferes with a comparison, as it would account for a difference in the results. From the careful mode in which M. Chomel has for a long period noted the cases of typhoid fever falling under his care, he would probably be enabled to furnish a table of the causes of deaths of a large number treated by the ordinary plan, with which a more legitimate comparison might be drawn. Although M. Chomel thinks that the facts are at present to a certain degree favorable to this mode of treatment, yet he repeats that they are not sufficient to establish clearly its efficacy. The striking contrast in 1831 and 1832, where it was not one in ten, or might be said to have been one in nineteen, and that in 1833-34 when it was raised to a third, should induce caution in deciding in favour of, or against, this remedy. More success having attended this treatment than any other, he is induced to persevere. This subject appears to have been discussed at the last meeting of the British Association in Dublin, in the Medical section. Dr. Graves stated that, owing to Dr. Reid's suggestion, he was induced to try it in 1832 in fever; he does not appear to have given the remedy throughout the course of the disease, but merely during the stage of prostration. "When the early stage of fever is passed, when all general and local indications have been fulfilled, when there is no complication with local disease, when the patient lies sunk and prostrated, when restlessness, low delirium, and more or less derangement of sensibility is present, when the body is covered with maculæ, and when the secretions from the skin and mucous membranes give evidence of a depraved state of the fluids, it is then that the chloride of sodium may be prescribed with the greatest advantage. The mode in which it acts I will not pretend to explain; it is sufficient to say, that there is no remedy from which, in such cases, such unequivocal benefit is derived. It operates energetically, though not very rapidly, in controlling many of those symptoms which create most alarm. It seems to counteract the tendency to tympanitis, to correct the fætor of the excretions, to prevent collapse, to promote a return to a healthy state of the functions of the skin, bowels, and kidneys; in fact, it seems admirably calculated to meet most of the bad effects of low putrid fever."

Dr. Stokes has also found that the use of this remedy was followed "by the most satisfactory results. It gradually but steadily removed all the bad symptoms, and in all cases the patients had most favorable convalescences." From fifteen to twenty drops were given in an ounce of water every four hours; the dose was never increased beyond this quantity; in all cases it had a fair trial, but was never continued longer than six or seven days. Wine, stimulants, and nutriment were given with it, according to the exigencies of the case. No statistical details of any sort are given, but it is stated by Dr. Graves that he has employed it in many hundred cases of fever.*

A chapter on the nature of typhoid fever concludes the volume, and is an excellent specimen of M. Chomel's style. There is a clearness in the statement of the arguments, and an amplitude of illustration, which make us regret that we must confine ourselves to a mere outline, instead of translating the whole.

Having successively examined the anatomical changes, symptoms, causes, and treatment of typhoid fever, there remains the investigation of its nature; or, if that is not capable of a complete solution, the order and importance of the anatomical lesions must at least be determined, and the diseases with which it is at all analogous. These

* Proceedings of the medical section of the British Association at Dublin, in August 1835. Dublin Journal of Medical and Chemical Science, September, 1835.

lesions have been divided into two groups: the constant, which includes diseases of the intestinal follicles and mesenteric glands, and the accidental, as inflamed conditions of various organs, only found occasionally, and not peculiar to fever. No one versed in pathology now considers this disease to be gastritis or gastro-enteritis; but the partisans of the same school who once affirmed this, change the name, and designate it follicular enteritis. In order to decide this point, several questions must be settled: 1. Is this follicular disease an inflammation? The redness, tumefaction, ulceration, suppuration, &c. of both follicles and glands indicate inflammation. 2. Is there any relation between the severity of the diseased changes and the symptoms? Experience proves that there is not, for that the most severe typhoid symptoms may exist with very slight disease of the follicles; whilst, on the other hand, very extensive ulcers may be present without producing serious symptoms. 3. Is the disease of the follicles constant? M. Chomel has never met with an exception; MM. Louis and Andral have seen some few cases in which all the symptoms of typhoid fever were present, without the peculiar lesion. 4. Are these anatomical changes primary or secondary? Thus, an inflammation is secondary when there is a morbid condition of which it is the consequence: it is primary when it alone constitutes the whole disease. If the changes in the follicles are examined, it is found that the disease is confined to these isolated patches of mucous membrane, and that the intervening portions are healthy. If we consider other cases of inflammations thus disseminated, we shall recognize differences between them and other inflammations. Thus, measles, smallpox, scarlet fever, nettle-rash, chicken-pox, boils, rheumatism, buboes of the plague, secondary syphilitic symptoms, abscesses after inflammations, &c., agree together in several points in which they differ from simple inflammations. (1.) There is a unity in each of these affections, however different in extent, situation, &c. in different cases. (2.) They cannot be artificially produced, like common inflammation. (3.) Antiphlogistics do not shorten their duration, and frequently do not diminish their activity. (4.) There is generally one termination peculiar to each. (5.) They apparently depend on specific causes. This is evident in many and highly probable in all. The causes are not similar to those which produce common inflammations. (6.) Are these inflammations primary or secondary? The bubo of syphilis and of the plague, the eruption of variola, scarlatina, &c., do not constitute the disease, but are only phænomena. This is expressed by variolæ sine variolis, morbilli sine morbillis, &c. of the nosologists. Numerous abscesses after inflammation, urticaria from indigestible food, and intermittents, are also secondary. It is generally admitted, finally, that rheumatism, boils, &c. are the expression merely of a morbid condition or diathesis. A special morbid condition, it may be said, must precede common inflammation, but this temporary modification of the economy is very different from that organic condition under the influence of which rheumatic inflammations, for instance, take place for months together in various parts of the body, having everywhere the same characters, and therefore depending on one cause; representing but one disease, although multiplied, and capable of suspension.

If these disseminated phlegmasiæ have distinct characters, depend on specific causes, and are the expression only of a diseased condition of body, as is proved in some, and is probable in others; and if, consequently, they occupy only a secondary place in the diseases in which they are observed, it is just to conclude that inflammation of the follicles of the intestines, from the fact alone that it is disseminated, is also only a secondary phenomenon of fever, that it does not constitute the point of departure for all the symptoms. If it is recollected also, 1st, that there is no relation between the extent of the lesion and the severity of the symptoms; and, 2dly, that the lesion has been found absent in those who have had the symptoms, it is more evident still that typhoid fever does not consist essentially in inflammation of the follicles; but that this inflammation is one of the phænomena of the disease; that it is secondary, like the majority of the disseminated inflammations, that it may be compared in its pathogenic value, not to the pustules of the smallpox of which the number is in proportion to the danger, but rather to the bubo of the plague. After having diminished the importance of the follicular disease in typhoid fever, it is necessary to repeat that its value as a characteristic lesion is very great; that, if it is not constant in the rigorous acceptation of the term, it is very rarely absent, and that there is no instance of this lesion in a patient who did not present the symptoms of typhoid fever. What then is the primary lesion? When anatomy fails in detecting any appreciable change, or when the lesions discovered will not explain the symptoms, we are obliged to admit some hidden change in the nervous system or in the fluids. M. Chomel is inclined to the belief that the primary impression must be on the blood, as impressions on the nervous system generally show themselves without fever, and produce no change appreciable after death. Here the absence of diseased appearances is a rare exception, and fever is intense: it may therefore be concluded, that the impression was not on the nervous system. On the other hand, although no change peculiar to this disease can be detected in the fluids, yet its analogy with variola, rubeola, scarlatina, plague, &c., in which there is manifestly infection of the fluids, points to a similar cause. If contagion was proved, many symptoms would be explained, such as the development of the disease at a particular period of life; its attacking but once; the want of proportion between the lesions and the symptoms; the absence of the lesion; and the little influence of antiphlogistics. But this question is still undecided.

This chapter concludes the volume. The complete analysis which we have given of it will be a sufficient proof of our estimation of the work. It is less elaborate than the standard production of the indefatigable and philosophic M. Louis on the same subject, whose plan M. Chomel has to a great extent adopted, but without sacrificing the independence of his own observations, which he has clothed in a more generally attractive form. The volumes of M. Louis will continue to be studied as a valuable book of reference, but the book which we have just closed will be more frequently read through.

---

